# Impact of a Dedicated Teaching Attending Experience on a Required Emergency Medicine Clerkship

**DOI:** 10.5811/westjem.2019.11.44399

**Published:** 2019-12-18

**Authors:** Todd A. Guth, Michael C. Overbeck, Kelley Roswell, Tien T. Vu, Kayla M. Williamson, Yeonjoo Yi, William Hilty, Jeff Druck

**Affiliations:** * University of Colorado School of Medicine, Department of Emergency Medicine, Aurora, Colorado; † University of Colorado School of Medicine, Department of Pediatrics, Aurora, Colorado; ‡ Saint Mary’s Medical Center, Department of Emergency Medicine, Grand Junction, Colorado

## Abstract

**Introduction:**

One published strategy for improving educational experiences for medical students in the emergency department (ED) while maintaining patient care has been the implementation of dedicated teaching attending shifts. To leverage the advantages of the ED as an exceptional clinical educational environment and to address the challenges posed by the rapid pace and high volume of the ED, our institution developed a clerkship curriculum that incorporates a dedicated clinical educator role – the teaching attending – to deliver quality bedside teaching experiences for students in a required third-year clerkship. The purpose of this educational innovation was to determine whether a dedicated teaching attending experience on a third-year required emergency medicine (EM) clerkship would improve student-reported clinical teaching evaluations and student-reported satisfaction with the overall quality of the EM clerkship.

**Methods:**

Using a five-point Likert-type scale (1 - poor to 5 - excellent), student-reported evaluation ratings and the numbers of graduating students matching into EM were trended for 10 years retrospectively from the inception of the clerkship for the graduating class of 2009 through and including the graduating class of 2019. We used multinomial logistic regression to evaluate whether the presence of a teaching attending during the EM clerkship improved student-reported evaluation ratings for the EM clerkship. We used sample proportion tests to assess the differences between top-box (4 or 5 rating) proportions between years when the teaching attending experience was present and when it was not.

**Results:**

For clinical teaching quality, when the teaching attending is present the estimated odds of receiving a rating of 5 is 77.2 times greater (p <0.001) than when the teaching attending is not present and a rating of 4 is 27.5 times greater (p =0.0017). For overall clerkship quality, when the teaching attending is present, the estimated odds of receiving a rating of 5 is 13 times greater (p <0.001) and a rating of 4 is 5.2 times greater (p=0.0086) than when the teaching attending is not present.

**Conclusion:**

The use of a dedicated teaching attending shift is a successful educational innovation for improving student self-reported evaluation items in a third-year required EM clerkship.

## INTRODUCTION

The emergency department (ED) is arguably the richest clinical teaching laboratory for medical students in medical education today, secondary to the high volume of patients with a broad range of undifferentiated complaints, and varying need for evaluation, stabilization, and diagnosis.^1^,^2^ Limited time, regular interruptions, and a lack of institutional rewards for education, as well as other barriers, contribute to the challenges of clinical teaching.^1^–^3^ The education of medical students within the ED demands a successful balance between providing high quality, efficient medical care to patients while creating outstanding educational experiences for learners.^2^–^4^ Currently, more than half of the medical schools in the United States require students to rotate through the ED during their undergraduate medical clerkships.^5^ Most of the required clinical emergency medicine (EM) clerkships take place in the fourth year of medical school; however, many schools offer clerkships in the third year. This dichotomy of required EM clerkships has driven the creation of educational curricula focused on both third-year and fourth-year experiences in EM.^6^–^8^ One published strategy for improving educational experiences for medical students in the ED while maintaining patient care has been the implementation of dedicated teaching attending shifts.^9^ Restructuring care teams with a focus on increased faculty supervision has improved trainee experiences and improved patient outcomes without increasing length of stay.^10^,^11^

To leverage the advantages of the ED as an exceptional clinical educational environment and to address the challenges posed by the rapid pace and high volume of the ED, our institution developed a clerkship curriculum that incorporates a dedicated clinical educator role, the teaching attending, to deliver quality bedside teaching experiences for students in a required third-year EM clerkship. This educational innovation is described below along with an analysis of student-reported evaluation items. The overall goal of the implementation of the teaching attending presence was to create an outstanding educational experience for students rotating on the required third-year EM clerkship. The primary objectives were to improve clinical bedside teaching evaluations and overall quality of the EM clerkship as assessed through student-reported evaluation ratings.

## METHODS

Third-year students are placed at one of three clinical sites for their required EM third-year clerkship: a quaternary care, university adult hospital; a quaternary, freestanding children’s hospital; or a regional community hospital. In any given academic year, approximately 50% of students rotate at the university adult hospital, approximately 30% of students rotate at the children’s hospital, and 20% of students rotate at the regional community hospital. Beginning with the graduating class of 2010, up to four teaching attending shifts, included in the total number of clinical shifts (typically seven shifts), were implemented at each of the three clinical sites. For the remaining ED shifts, students were distributed into shifts with regularly scheduled ED attendings. There was a temporary loss of the teaching attending experience for a 16-month period at a single site—the university adult hospital—from April 2013 through August 2014 primarily affecting the graduating classes of 2015 and 2016.

The teaching attending experience created a new clinical shift for faculty to work specific educational shifts with third-year medical students. The teaching attending experience was implemented with the objectives of improving clinical bedside teaching, increasing the direct observation of students’ clinical skills, providing medical documentation review, and allowing direct access to attendings for supervision of procedures and facilitation of the inter-professional aspects of patient care. The teaching attendings were selected faculty at each clinical who received training on the learning goals and objectives of the clerkship and on bedside teaching skills. Typically, two medical students were paired with a single teaching attending during a shift.

Using medical student-reported items gathered through the School of Medicine evaluation office, we tracked evaluation ratings for the EM clerkship since the inception of the EM clerkship for the graduating class of 2009 through and including the graduating class of 2019. The Colorado Combined Institutional Review Board granted an exempt approval for the retrospective evaluation of these medical student self-reported items. Using a five-point Likert-type scale (1 - poor to 5 - outstanding), medical students were asked anonymously through the School of Medicine evaluation office to rate the EM clerkship in terms of 1) “What was the quality of clinical teaching in this clerkship?” and 2) “What was the overall quality of the clerkship?” We collected survey responses from 1315 students over the 10-year study period with approximately 125–170 students completing the EM clerkship each academic year. Students rotating on the EM clerkship are required to fill out an evaluation for the clerkship, including these two evaluation items, as a requirement for completion of the clerkship.

We assessed the overall trends of the percentage of individual item ratings, means, and top-box proportions (the sum of ratings of 4 or 5 for the evaluation items) for each evaluation item by graphical inspection across all three clinical sites. The total number of students rotating in the clerkship and the number of students matching in EM upon graduation were also tracked. Despite the university adult hospital site being the only site to temporarily lose the teaching attending experience, primary analyses regarding estimated odds for the frequency of ratings and top-box proportions comparisons were reported across all three sites. We used a multinomial logistic regression to evaluate whether the introduction of a teaching attending experience impacted the overall student-reported evaluation ratings. Ratings for the two evaluation items were the primary outcome of interest and the presence of the teaching attending experience was the independent variable.

Additionally, as a surrogate for an outstanding experience, top-box ratings were computed as the sum of ratings of 4 or 5 for the evaluation items. Evaluation ratings for the graduating classes of 2013, 2015, and 2017 were selected for top-box comparisons to allow for a one-year period of washout following the initial implementation of the teaching attending experience for the class of 2012 and the re-implementation of the teaching attending experience after its loss for the class of 2015. We used two sample proportion z-tests to assess the differences between top-box (4 or 5 ratings) proportions between years. P-values were determined for the three comparisons. P-values were unadjusted for multiple comparisons.

## RESULTS

**Figure 1:**
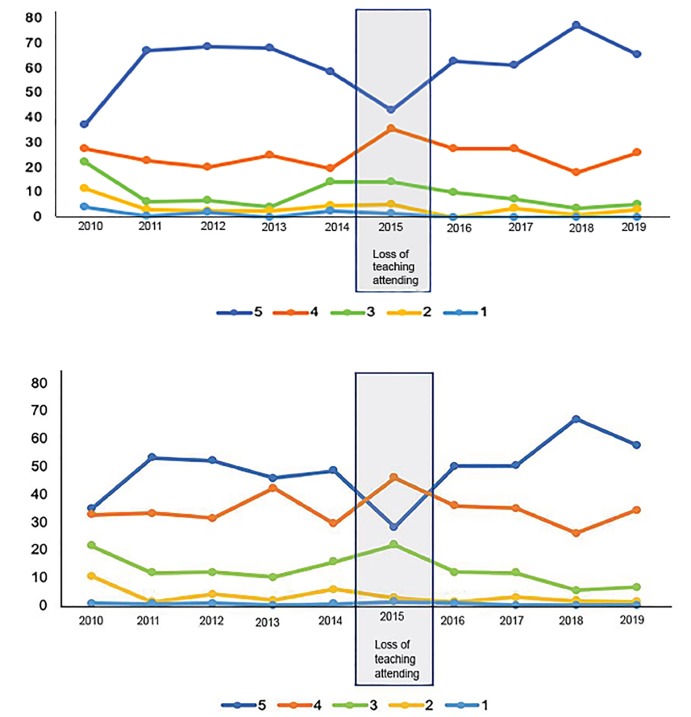
Percentage of ratings for evaluation items related to quality of the clinical teaching and overall clerkship quality across graduating class.

The percentage of individual ratings (1 - poor to 5 - outstanding) for each evaluation item across all three sites for 1) quality of clinical teaching and 2) overall quality of the clerkship are shown in Figure 1 across the 10 years of available data. Table 1 displays a numeric overview of the means and standard deviations for each evaluation item over time, as well as the total number of students rotating in the EM clerkship and the number of students matching into EM residency during the study period. The temporary loss of the teaching attending experience occurred for a total of 16 months primarily affecting the graduating class of 2015 (12 months) with a lesser impact on the graduating class of 2016 (four months).

For the evaluation item related to clinical teaching quality, when the teaching attending was present, the estimated odds of receiving a rating 5 was 77.2 times greater than when the teaching attending was not present (p <0.001; 95% confidence interval [CI], 9.86–603.35), and similarly, the estimated odds of receiving a rating 4 was 27.5 times greater than when the teaching attending was present than not (p = 0.0017; 95% CI, 3.46–218.58). For the evaluation item related to overall clerkship quality, when the teaching attending was present, the estimated odds of receiving a rating 5 was 13.0 times greater than when the teaching attending was not present (p <0.001; 95% CI, 3.78–44.57), and the estimated odds of having a rating of 4 was 5.3 times more likely when a teaching attending was present than not (p = 0.0086; 95% CI, 1.53–18.22).

**Figure 2:**
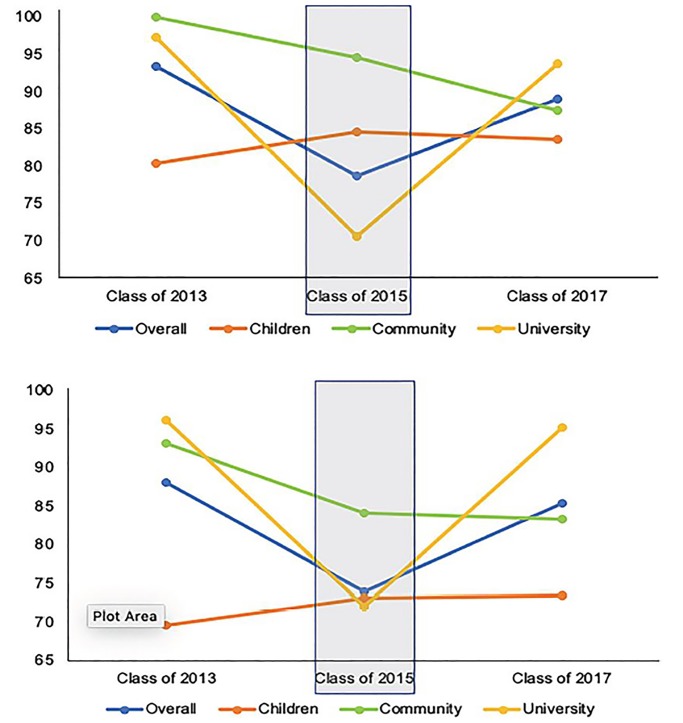
Top-box proportions of ratings for the classes of 2013, 2015, and 2017 for each clinical site and overall for quality of clinical teaching and overall clerkship quality. Overall – composite results at all three clinical sites. Children – quaternary care, freestanding children’s hospital. Community – regional community hospital. University –quaternary care, university adult hospital.

For clinical teaching quality, there was a significant difference in top-box ratings between the graduating classes of 2013 and 2015 (Table 2; p<0.001) as well as a significant difference between top-box ratings for the classes of 2015 and 2017 (Table 2; p = 0.029). There was no significant difference between the classes of 2013 and the class of 2017 suggesting that the removal of the attending for the class of 2015 at the university adult hospital had a negative impact in top-box ratings during this academic year (Table 2). Similarly, for overall clerkship quality, there was a significant difference in top-box ratings between the graduating classes of 2013 and 2015 (Table 1; p = 0.002) as well as a significant difference between top-box ratings for the classes of 2015 and 2017 (Table 2; p = 0.025). There was no significant difference between the graduating classes of 2013 and 2017 suggesting that the removal of the attending for the class of 2015 had a negative impact in top-box ratings during this year (Table 2). Top-box ratings for the graduating classes of 2013, 2015, and 2017 across all three clinical sites and as a composite are represented graphically in Figure 2.

## DISCUSSION

With the exception of the temporary loss of the teaching attending experience for some of the learners in the graduating classes of 2015 and 2016, the addition of a teaching attending experience to the EM clerkship for the graduating class of 2011 has had a significant positive impact on student reported evaluation items through the class of 2019. The temporary loss of the teaching attending experience, primarily for the graduating classes of 2015, at the adult university site and its resultant negative association on the student-reported evaluation items also supports the ongoing effectiveness and impact of the teaching attending experience. The data we provide here reinforces the inference that dedicated teaching attending experiences have a positive association on student-reported evaluation items. Moreover, this dataset adds to the current body of evidence by providing a larger breadth of data using 10 years of evaluation data from over 1000 medical student respondents.

Teaching attendings receiving dedicated training that develops bedside teaching skills and provides clear educational expectations can impact student perceptions of their educational experience. The commitment to provide a teaching attending experience for medical students represents a substantial investment in medical student education in terms of attending physician time and departmental resources. Medical students clearly appreciate these investments into their education by rating the EM clerkship as outstanding.

## LIMITATIONS

The primary outcome of student-reported satisfaction is low-level evidence for the impact of an educational intervention based upon the Kirkpatrick level of evidence model,^12^ and, therefore, is the main limitation of this study. Moreover, these two evaluation items have not been previously validated. More robust outcomes on the impact of the educational interventions on the Kirkpatrick model should be considered. These additional data might include impacts of student clinical skills assessments, patient throughput, patient satisfaction, and possibly patient clinical outcomes. We did include data related to the numbers of students matching into EM residency upon graduation, but we recognize that the numbers of students matching into any residency training program is influenced by a multitude of factors beyond the presence or absence of a teaching attending experience. While the graduating class of 2016 did have the lowest numbers of students choosing to enter EM residency programs over the 10-year period, we could not determine whether this was a direct causative effective of teaching attending loss during their EM clerkship.

Second, because these data were analyzed in a retrospective fashion without a specific experimental design, there were multiple, confounding variables that could have influenced these student-reported evaluation items. Third, while collected at three different clinical sites, this data represents student-reported evaluation ratings for an EM clerkship experience at a single institution. Fourth, while our data represents improvements in student evaluation items, the impact of the teaching attending experience may not translate into actual student learning improvements and true educational value. Nevertheless, the range of student evaluation items across multiple years of data since the implementation of the teaching attending experience, during the hiatus of the teaching attending experience, and the re-implementation of the teaching attending experience make this dataset intriguing and relevant.

## CONCLUSION

Despite the limitations, the evaluation items related to overall clerkship quality and clinical teaching quality represent a reasonable surrogate of the overall educational experience for medical students on a required EM clerkship. Based on the analysis of the reported evaluation items, the use of a dedicated teaching attending experience demonstrates an association with improved clinical bedside teaching evaluations and an improved rating for the overall quality of the EM clerkship. The teaching attending experience may be a successful and sustainable educational innovation for EDs willing to make the commitment to create a teaching attending experience for medical students.

## Figures and Tables

**Table 1:** Mean student-reported satisfaction with standard deviations for each evaluation item, total number of respondents, and number of students matching into emergency medicine residency programs upon graduation across graduation year.

Graduation year	Clinical teaching quality Mean-SD	Overall clerkship quality Mean-SD	Total number of respondents	Number of students matching into emergency medicine residency
Class of 2010	3.79 – 1.02	3.67 – 1.16	135	14
Class of 2011	4.32 – 0.67	4.13 – 0.79	155	17
Class of 2012	4.51 – 0.89	4.35 – 0.88	150	19
Class of 2013	4.60 – 0.69	4.32 – 0.73	168	16
Class of 2014	4.29 – 0.68	4.19 – 0.95	153	12
Class of 2015*	4.14 – 0.84	3.97 – 0.86	146	14
Class of 2016*	4.50 – 0.68	4.29 – 0.81	142	9
Class of 2017	4.37 – 0.84	4.33 – 0.83	137	15
Class of 2018	4.72 – 0.62	4.58 – 0.74	127	20
Class of 2019	4.55 – 0.73	4.48 – 0.69	137	17

* There was a loss of the teaching attending at the tertiary referral university adult hospital for 12 months for the Class of 2015 and four months for the Class of 2016.

*SD*, standard deviation.

**Table 2:** Top-box proportions and comparisons for clinical teaching quality and overall clerkship quality for the graduating classes of 2013, 2015, and 2017.

	Class of 2013	Class of 2015	Class of 2017
Clinical teaching quality: top-box proportions	0.93	0.79	0.84
Comparisons		p-value	
Class of 2013 to Class of 2015		<0.001	
Class of 2015 to Class of 2017		0.029	
Class of 2013 to Class of 2017		0.25	
Clerkship quality: top-box proportions	0.88	0.74	0.85
Comparisons		p-value	
Class of 2013 to Class of 2015		0.002	
Class of 2015 to Class of 2017		0.025	
Class of 2013 to Class of 2017		0.6	
